# Three new cavernicolous species of dragon millipedes, genus *Desmoxytes* Chamberlin, 1923, from southern China, with notes on a formal congener from the Philippines (Diplopoda, Polydesmida, Paradoxosomatidae)

**DOI:** 10.3897/zookeys.185.3082

**Published:** 2012-04-23

**Authors:** Sergei I. Golovatch, Youbang Li, Weixin Liu, Jean-Jacques Geoffroy

**Affiliations:** 1 Institute for Problems of Ecology and Evolution, Russian Academy of Sciences, Leninsky pr. 33, Moscow 119071, Russia; 2 College of Life Science, Guangxi Normal Univeristy, Guilin, Guangxi Province 541004, China; 3 College of Natural Resources and Environment, South China Agricultural University, Guangzhou City, Guangdong Province 510642, China; 4 Muséum national d’Histoire naturelle, Département Ecologie & Gestion de la Biodiversité, UMR 7204 du CNRS, Equipe EVOLTRAIT, avenue du Petit Château 4, Brunoy 91800, France

**Keywords:** Millipede, cave, *Desmoxytes*, new species, key, China

## Abstract

The large Southeast Asian genus *Desmoxytes* is slightly rediagnosed. A number of troglomorphic, most likely troglobitic, species occur in southern China. A key is provided to all 10 *Desmoxytes* spp. currently known from China, including three new presumed troglobites: *Desmoxytes eupterygota* **sp. n.** from Hunan Province, as well as *Desmoxytes spinissima* **sp. n.** and *Desmoxytes lui* **sp. n.** from Guangxi Province. “*Desmoxytes” philippina* Nguyen Duc & Sierwald, 2010, from the Philippines, is formally removed from *Desmoxytes*, but not assigned to another genus. It probably belongs in a new genus in the subfamily Australiosomatinae, tribe Antichiropodini, close to the Bornean *Euphyodesmus* Attems, 1931 and *Borneochiropus* Golovatch, 1996.

## Introduction

Originally, the name “dragon millipedes” was proposed as a vernacular name to distinguish *Hylomus draco* Cook & Loomis, 1924, a Chinese species showing unusually spiny paraterga, Apparently, Cook and Loomis (1924) were so impressed by this feature that they placed the species not only in a new genus, but also created a new family. However, Jeekel (1968, 1980) demonstrated that actually several Southeast Asian species share this peculiar character, referring to *Hylomus* as a synonym of the slightly older *Desmoxytes* Chamberlin, 1923, assigning the latter genus to the tribe Orthomorphini, and sinking Hylomidae under Paradoxosomatidae. Golovatch and Enghoff (1994), followed by Nguyen Duc et al. (2005), adopted the notion “dragon millipedes” as a fairly compact and homogeneous group of species composing the genus *Desmoxytes*, tribe Orthomorphini, subfamily Paradoxosomatinae, in which the paraterga are wing- or antler-shaped, or even spiniform. Since the genus was based only on a couple of equivocal apomorphies, the generic diagnosis of *Desmoxytes* as presented by Golovatch and Enghoff (1994) remained rather shaky whilst their cladistic analysis showed no meaningful resolution.

After Mesibov (2006) had described *Desmoxytoides*, a monobasic genus from eastern Australia which also demonstrates antler-like paraterga, the “dragon millipedes” again became a vernacular name meaningless from a taxonomic or evolutionary viewpoint (Enghoff et al. 2007, Nguyen Duc and Sierwald 2010). Even though Mesibov (2006) had failed to assign *Desmoxytoides* to a tribe or subfamily, due to the presence of a thick solenomere lacking a nearby solenophore the type species *Desmoxytes hasenpuschorum* Mesibov, 2006 shows, it clearly represented a different subfamily, Australiosomatinae, albeit rather untypical of it because male legs 1 were devoid of adenostyles. This alone suggested multiple origins of conspicuously hypertrophied paraterga within Paradoxosomatidae. Parallelisms have been reconfirmed and further extended by Golovatch and Stoev (2010) who described a similarly spinigerous species of *Tectoporus* Carl, 1914 (Tectoporini, Paradoxosomatinae) from Papua New Guinea. Based on gonopod features alone, these authors also suggested placing provisionally both *Desmoxytoides* and *Desmoxytes philippina* Nguyen Duc and Sierwald, 2010, the latter species from the Philippines, into the Australasian tribe Antichiropodini (Australiosomatinae). This agrees far better with biogeographical evidence as well, because Borneo supports still another few species in *Euphyodesmus* Attems, 1931 and *Borneochiropus* Golovatch, 1996, both latter genera again in Antichiropodini, which show spine-shaped paraterga (Golovatch 1996). Furthermore, Golovatch and Stoev (2010) have proposed to restrict the usage of the term “dragon millipedes” solely to the genus *Desmoxytes*, a group still fairly well diagnosed earlier by Golovatch and Enghoff (1994) despite certain shortcomings (Mesibov 2006; Enghoff et al. 2007). An updated, only slightly modified diagnosis will be provided below.

In other words, antler-like or spiniform paraterga must have evolved independently at least in three different lineages of Paradoxosomatidae representing three tribes and two subfamilies: *Desmoxytes* (Orthomorphini, Paradoxosomatinae), or “dragon millipedes” in the proper sense; *Tectoporus* (Tectoporini, Paradoxosomatinae); as well as *Desmoxytoides*, *Euphyodesmus*, *Borneochiropus* and *“D.” philippina* (see below) (Antichiropodini, Australiosomatinae).

The genus *Desmoxytes* s. str. is currently represented by 26 species ranging from southern China in the North to about the middle of Malay Peninsula within both Thailand and Malaysia in the South. Most of the species diversity is found in Vietnam (9 species), followed by Thailand (8), southern China (7) and Myanmar (2), while neither Cambodia nor Laos has hitherto been known to support any *Desmoxytes*, even the sole pantropical congener, *Desmoxytes planata* (Pocock, 1895). Most of the species are quite local in distribution: the few which have been reported from southern China tend to be restricted to a single locality each. This alone suggests that many more new species of *Desmoxytes* will be revealed in the future.

Several *Desmoxytes* species show remarkably bright, apparently aposematic live colorations ranging from intense carmine to pink or purple-pink (see review in Enghoff et al. 2007). One such species, *Desmoxytes purpurosea* Enghoff, Sutcharit and Panha, 2007, comes from a small karstic area in northern Thailand, originally probably a cave, but later having its roof collapse, resulting in the present cavernous mountain with high humidity throughout (Enghoff et al. 2007). However, all three heretofore known troglomorphic, most likely even troglobitic congeners, *Desmoxytes longispina* (Loksa, 1960), *Desmoxytes scutigeroides* Golovatch, Geoffroy and Mauriès, 2010 and *Desmoxytes scolopendroides* Golovatch, Geoffroy and Mauriès, 2010, are pallid to pale brownish in coloration, being confined to karst caves in Guangxi Province (Golovatch et al. 2010). Both latter species come from Mulun Karst which probably contains the richest cave fauna not only in China, but even globally (Deharveng et al. 2008). Epigean congeners from China have only been recorded in Jiangxi (*Desmoxytes draco* (Cook and Loomis, 1924)), Guangxi (*Desmoxytes cornutus* (Zhang and Li, 1982) and *Desmoxytes minutubercula* (Zhang, 1986)) and Yunnan provinces (*Desmoxytes planata*). At least the latter species shows an intense red, aposematic live coloration which fades out completely during preservation in alcohol, whereas the coloration in the remaining trio is light, yellowish to light brown in alcohol (Zhang and Li 1982, Zhang 1986, Golovatch and Enghoff 1994). Another interesting observation is that the few species of *Desmoxytes* which have spiniform paraterga are all confined to Guangxi Province, several being presumed troglobites.

Generally speaking, the family Paradoxosomatidae, one of the largest in Diplopoda as a whole, contains surprisingly few cavernicoles. It is only the large genus *Desmoxytes* that appears to harbourseveraltroglomorphic species, with caves in southern China hosting virtually all of them.

The present note is devoted to descriptions of three new cave-dwelling *Desmoxytes* from southern China, i.e. two more from Guangxi Province, as well as the first species from Hunan Province. A key is also given to all ten species of *Desmoxytes* known to occur in China. In addition, a recent error concerning the identity of a formal congener from the Philippines is being corrected here.

The holotypes will be deposited in the collection of the Institute of Zoology, Chinese Academy of Sciences, Beijing, China (IZAS), with paratypes to be housed in the collections of the South China Agricultural University, Guangzhou, China (SCAU), Guangxi Normal University,Guilin, China (GNUG), Zoological Museum, University of Moscow, Russia (ZMUM), and Muséum national d’Histoire naturelle, Paris, France (MNHN).

A dynamic web page for each taxon name mentioned in the paper is generated on the fly by the Pensoft Taxon Profile tool (see Penev et al. 2010). All species descriptions are automatically exported at the time of publication to a wiki platform (www.species-id.net) through the Pensoft Wiki Convertor (see Penev et al. 2011, Stoev and Enghoff 2011).

## Taxonomic part

### Desmoxytes

Chamberlin, 1923

http://species-id.net/wiki/Desmoxytes

#### Diagnosis.

A genus of medium-sized to larger (up to 43 mm long) Orthomorphini with 20 segments and conspicuously enlarged and elevated paraterga (antler-like, wing-shaped or spiniform). Metaterga often granulate, tuberculate and/or spiculate. Certain male femora (5, 6, 7 and/or 9) often inflated ventrally. A setose central lobe or a paramedian pair of setose tubercles between male coxae 4 present.

Gonopods with rather short, subcylindrical, distoventrally setose coxae. Telopodites mostly suberect, only seldom subfalcate. Prefemoral (= densely setose) portion from 1/3 to 1/2 as long to nearly as long as femorite, the latter not twisted, at most only slightly enlarged distad and devoid both of a mesal groove/hollow and any processes. Seminal groove running entirely mesally to pass onto a usually shortened solenomere, the latter mostly sheathed by a usually condensed, rather simple solenophore, much shorter than femorite, composed of a smaller lamina medialis and a larger lamina lateralis. Solenophore demarcated from femorite by a clear-cut sulcus or cingulum at base, poorly or strongly set off from base of solenomere.

#### Type species.

*Desmoxytes coniger* Chamberlin, 1923

#### Species composition.

Currently 29 described species.

#### Remarks.

The above diagnosis largely repeats that given by Golovatch and Enghoff (1994). Here it only emphasizes variation in relative lengths of the femoral and solenophore parts of the gonopod telopodite.

### Species descriptions

#### Desmoxytes eupterygota sp. n.

urn:lsid:zoobank.org:act:F7B1C583-5E3C-4980-8EB1-7BB9D3BFB5F3

http://species-id.net/wiki/Desmoxytes_eupterygota

Figs 1 2

##### Holotype.

♂(IZAS), China, Hunan Prov., Chenzhou City, Linwu County, Tianhe, Cave 1, 500 m a.s.l., 19.VI.2009, leg. Tian Mingyi & Xue Zhihong (CHIhn09-LWX02).

##### Paratypes.

1♂(SCAU), 1 ♀ (ZMUM), same locality, together with holotype. 1 ♂ (MNHN JA 130), 1 ♂juv., 1 ♀ juv., 1 fragment (SCAU), 1 ♀ juv. (GNUG), same county, Changshali, Cave 1, 500 m a.s.l., 19.VI.2009, leg. Tian Mingyi & Xue Zhihong (CHIhn09-LWX03).

##### Name.

To emphasize the paraterga being true wings.

##### Diagnosis.

Differs in the paraterga being mostly wing-shaped, rather long and strongly curved, combined with a short gonopod femorite and a condensed solenophore, as well as ♂legs totally devoid of femoral humps. See also Key below.

##### Description.

Length ca 25–29 (♂) or 32 mm (♀), juveniles (♀ with 18 segments) up to 26 mm; width of proterga and metaterga+paraterga 1.8 and 2.0–2.3 (♂), or 2.3 and 2.8 mm (♀), respectively. Holotype 27 mm long, 1.8 and 2.3 mm wide on midbody pro- and metazona, respectively. Juveniles (♀ with 18 segments) up to 1.9 and 2.8 mm wide on midbody pro- and metazona, respectively.

**Figure 1.**
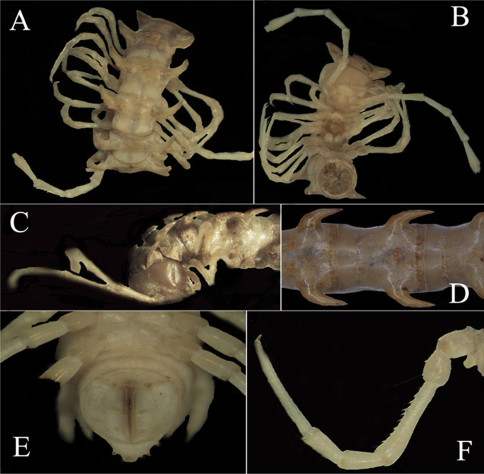
*Desmoxytes eupterygota* sp. n., ♂ paratype from near Tianhe. **A–C** anterior part of body, dorsal, ventral and lateral views, respectively **D** midbody segments, dorsal view **E t**elson, ventral view **F** midbody leg, front view.Photographed not to scale.

Body moniliform (Fig. 1D). Coloration of alcohol material (♂**,** ♀ and advanced juvenile instars) rather uniformly light grey-brownish to yellowish, anterior body part often a little darker brownish (Fig. 1A–C). In width, head << collum = segment 2 = 3 < 4 = 16; thereafter body gradually tapering towards telson. Front part of head densely setose, vertex bare, epicranial suture distinct. Antennae very long and slender, reaching back to segment 5 or 6 (♂) (Fig. 1A–C), or 4 (♀) dorsally, antennomeres 5 and 6 each with an apicodorsal compact group of bacilliform sensilla.

Tegument rather smooth and shining, both pro- and metazona very delicately microalveolate, metaterga finely shagreened and rugulose transversely, surface below paraterga finely shagreened. Collum with 3+3 small, but rather evident teeth in a row at front margin, behind it with 2+2 and 2+2 extremely small denticles, knobs or insertion points (setae invariably obliterated) in two transverse, often barely visible rows. Metatergum 2 with a pattern of similar, barely visible 2+2 and 2+2 knobs or insertion points in two transverse rows. Starting from metatergum 3, setation pattern entirely or nearly untraceable. Collum and all following metaterga with large, mostly subfalcate, wing-shaped, high paraterga (Fig. 1A–D) directed dorsolaterally and ending up clearly above dorsum on collum, as well as segments 2–7 and 17 & 18, remaining paraterga subhorizontal and about level with dorsum in ♂, but slightly lower and shorter in ♀ and juveniles. Paraterga with two indentations at front margin, starting from collum these becoming less distinct and nearly fully disappearing in segments 15 to 18. Paraterga 17–19 directed caudad, subspiniform (Fig. 1E). Stricture between pro- and metazona narrow only in a few anteriormost segments, thereafter much wider and only vaguely delimited, always smooth at bottom (Fig. 1D). Pore formula normal; ozopores inconspicuous, located about midway on ventral side of poriferous paraterga. Transverse sulcus evident on metaterga 3–18 (Fig. 1D). Pleurosternal carinae poorly developed in segments 2 and 3, absent from others (Fig. 1C). Epiproct (Fig. 1E) rather simple, dorsal subapical and, especially, lateral pre-apical papillae very distinct, tuberculiform. Hypoproct (Fig. 1E) subtrapeziform, caudal margin very slightly concave, setigerous cones at caudal edge very small, widely separated. Axial line missing.

**Figure 2.**
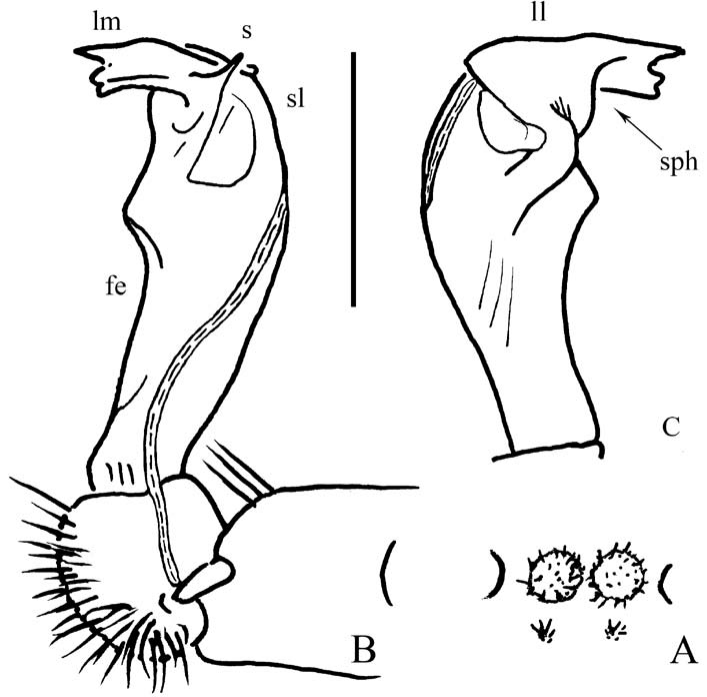
*Desmoxytes eupterygota* sp. n., ♂ paratype from near Tianhe. **A** sternal cones between coxae 4, ventral view **B** and **C** right gonopod, mesal and lateral views, respectively. Scale bar: 1.0 (**A**) and 0.5 mm (**B, C**). Designations: **fe** femorite **sph** solenophore **sl** solenomere **ll** lamina lateralis **lm** lamina medialis **s** spinule on **lm**

Sterna quite densely setose, cross-impressions faint. A paramedian pair of entirely separated, short, rounded, setose tubercles between ♂ coxae 4 (Fig. 2A). Legs (Fig. 1A, B, F) very long and slender, devoid of modifications, ca 3.2–3.5 (♂) or 2.5 (♀) times longer than midbody height.

Gonopods (Fig. 2B, C) short. Coxite rather short, subcylindrical, poorly setose distodorsally, about half as long as telopodite. Prefemoral (= densely setose) portion less than half as long as acropodite. Femorite (**fe**) quite stout, slightly enlarged distad, with seminal groove running entirely on mesal face, apically with a distinct sulcus demarcating a short, strongly condensed solenophore (**sph**). The latter distinguished by a parabasally spinigerous (**s**) and terminally poorly trifid lamina medialis (**lm**) and a simpler and somewhat larger lamina lateralis (**ll**). Solenomere (**sl**) similarly short, flagelliform, well separated at base from solenophore.

#### Desmoxytes spinissima sp. n.

urn:lsid:zoobank.org:act:805A9FDB-02DC-4449-93AE-5C514FD59D0E

http://species-id.net/wiki/Desmoxytes_spinissima

Figs 3 4

##### Holotype.

♂(IZAS), China, Guangxi Prov., Fuchuan County, Guanyuan, Cave Guanyuan Dong, 25.I.2012, leg. Li Youbang, Li Youting and Tang Kewen (CHIgx12-LYB04).

##### Paratype.

1 ♀ (GNUG), same locality, together with holotype.

##### Name.

To emphasize the extremely long and spiniform paraterga.

##### Diagnosis.

Differs in the paraterga being long and spiniform throughout, combined with a strongly condensed solenophore and both ♂ femora 6 and 7 being humped. See also Key below.

##### Description.

Length ca 27 (♂) or 36 mm (♀); width of pro- and metaterga together with paraterga 1.6 and 4.0 (♂) or 2.0 and 4.0 mm (♀), respectively.

**Figure 3.**
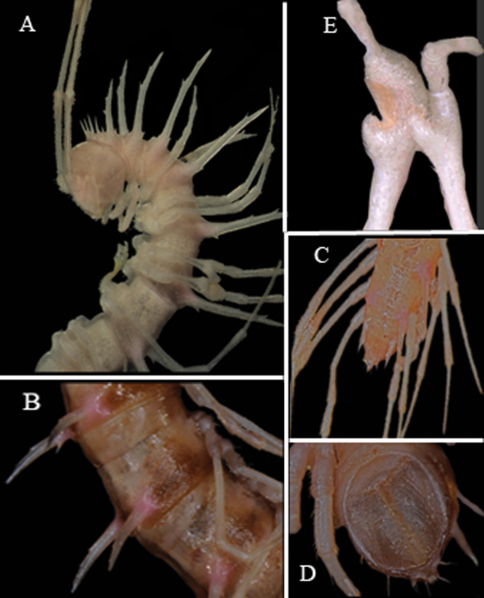
*Desmoxytes spinissima* sp. n., ♂ holotype. **A** anterior part of body, lateral view **B** midbody segments, lateral view **C** posterior part of body, dorsal view **D** telson, ventral view **E** femora 6 and 7, lateral view.Photographed not to scale.

Body strongly moniliform (Fig. 3A, B). Coloration of alcohol material rather uniformly light pink-brownish to nearly pallid, anterior body part a little darker, pinkish (Fig. 3). Antennomere 7 dark brown. In width, head << collum = segment 2 = 3 < 4 < 5 = 16; thereafter body gradually tapering towards telson. Head densely setose throughout, epicranial suture distinct. Antennae very long and slender, reaching back to segment 8 (♂) or 6 (♀) dorsally, antennomeres 5 and 6 each with an apicodorsal compact group of bacilliform sensilla.

Tegument rather smooth and poorly shining, prozona very delicately microalveolate, metaterga finely shagreened to microgranulate/microspiculate, surface below paraterga finely shagreened. Collum with 3+3 evident setigerous spines in a row at front margin (growing increasingly long laterad), behind it with 1+1 and 2+2 similar spinules in two transverse rows. Following metaterga with a pattern of similar 1+1 and 2+2 spinules in two transverse rows. Collum and all following metaterga with extremely long, straight, spiniform paraterga (Fig. 3A, B) directed more dorsally than laterally and ending up clearly above dorsum on collum and in segments 2–18; only paraterga 19 subhorizontal, about level with dorsum, directed clearly caudad and reaching behind until about midlength along telson. Paraterga ca 1.2–1.3 (♂) or 0.9 times (♀) as long as midbody height. Paraterga 1–18 with 2–3 evident indentations/spinules frontally (these growing increasingly inconspicuous towards telson) and a short, but evident tooth posteriorly at base. Stricture between pro- and metazona rather narrow and shallow, always smooth at bottom (Fig. 3B). Pore formula normal; ozopores inconspicuous, located just behind last indentation on ventral side of poriferous paraterga. Transverse sulcus missing. Pleurosternal carinae evident only in segments 2 and 3. Epiproct (Fig. 3C, D) rather simple, subapical and, especially, pre-apical papillae very distinct, finger-shaped. Hypoproct (Fig. 3D) subtrapeziform, caudal margin emarginate, setigerous cones at caudal edge small, but evident, widely separated. Axial line missing.

**Figure 4.**
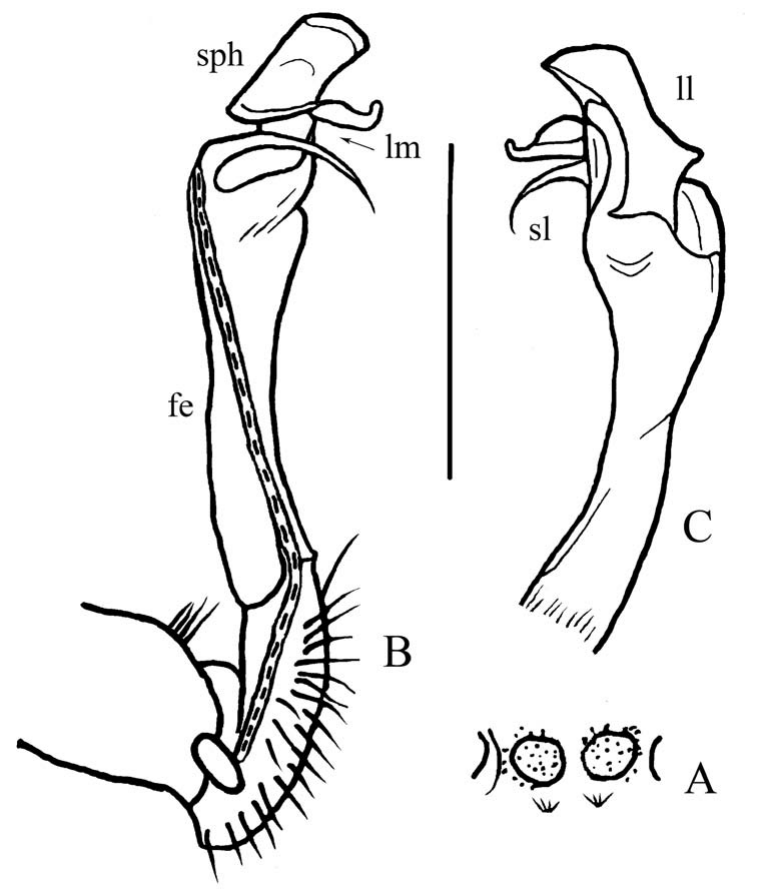
*Desmoxytes spinissima* sp. n., ♂ holotype. **A** sternal cones between coxae 4, ventral view **B–C** left gonopod, mesal and lateral views, respectively. Scale bar: 1.0 (**A**) and 0.5 mm (**B, C**). Designations: **fe** femorite **sph** solenophore **sl** solenomere **ll** lamina lateralis **lm** lamina medialis

Sterna quite densely setose, cross-impressions faint. A paramedian pair of entirely separated, short, rounded, setose tubercles between ♂ coxae 4 (Fig. 4A). Legs (Fig. 3A–C) extremely long and slender, > 4.0 (♂) or 3.2 (♀) times longer than midbody height. ♂femora 6 and 7 strongly inflated ventrally in distal quarter (Fig. 3E)

Gonopods (Fig. 4B, C) simple. Coxite rather short, subcylindrical, poorly setose distodorsally, about 1/3 as long as telopodite. Prefemoral portion (= densely setose) about half as long as acropodite. Femorite (**fe**) slender, elongate, only slightly enlarged distad, with seminal groove running entirely on mesal face, also with a distinct sulcus demarcating a short, strongly condensed solenophore (**sph**). The latter distinguished by a smaller, somewhat folded lamina medialis (**lm**) and a simpler and larger lamina lateralis (**ll**). Solenomere (**sl**) similarly short, flagelliform, rather faintly separated at base from solenophore.

#### Desmoxytes lui sp. n.

urn:lsid:zoobank.org:act:A0C24C82-1F34-437A-BCFA-C28A1E3BA72F

http://species-id.net/wiki/Desmoxytes_lui

Figs 5 6

##### Holotype.

♂(IZAS), China, Guangxi Prov., Yongfu County, Shangxiao, Cave Dachong Dong, 17.I.2012, leg. Li Youbang and Lu Shiyi (CHIgx12-LYB03).

##### Paratypes.

1 ♀ (GNUG), 1 ♀ juv. (MNHN JA 131), same locality, together with holotype.

##### Name.

To honour Mr Lu Shiyi, one of the collectors.

##### Diagnosis.

Differs in the paraterga being spiniform only until segment 5, combined with the gonopods being strongly condensed, ♂femora 6 humped. See also Key below.

##### Description.

Length ca 36 (♂), 43 mm (♀) or 40 mm (1 ♀ juvenile with 19 segments); width of pro- and metaterga together with paraterga 2.0 and 2.2 (♂), 3.2 and 3.4 mm (♀) or 3.0 and 3.1 mm (1 ♀ juvenile with 19 segments), respectively.

**Figure 5.**
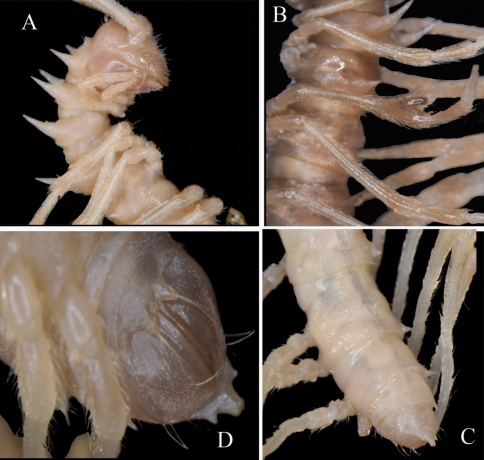
*Desmoxytes lui* sp. n., ♂ holotype. **A** anterior part of body, lateral view **B** body segments 5 to 8, lateral view **C** posterior part of body, dorsal view **D** telson, ventral view.Photographed not to scale.

Body strongly moniliform (Fig. 5A–C). Coloration of alcohol material rather uniformly light pink-brownish to nearly pallid, anterior body part a little darker, pinkish (Fig. 5). Antennomere 7 dark brown (even in the juvenile). In width, segment 3 = 4 < head < collum = segment 2 < 6 = 16; thereafter body gradually tapering towards telson. Head very densely setose throughout, epicranial suture distinct. Antennae extremely long and slender, reaching back to segment 9 (♂) or 7 (♀) dorsally, antennomeres 5 and 6 each with an apicodorsal compact group of bacilliform sensilla.

Tegument rather smooth and poorly shining, prozona very delicately microalveolate, metaterga finely shagreened to microgranulate/microspiculate, surface below paraterga finely shagreened to microgranulate. Collum with 6+6(7) evident setigerous spinules in a row at front margin, behind it with about 4+4 smaller spinules in an irregular transverse row.

Following metaterga with a pattern of similar, smaller, 3+3 and 3+3 spinules in two transverse rows, last row gradually growing up to 6+6 until segment 19. Collum and following metaterga 2–4 with straight, spiniform paraterga, about as long as body height in ♂, a little shorter in ♀ and juvenile (Fig. 5A–C), directed much more dorsally than laterally and ending up clearly above dorsum; paraterga 5 only slightly shorter than preceding ones; paraterga 6–18 contrasting short, coni- to dentiform; paraterga 19 subhorizontal, about level with dorsum, directed clearly caudad and reaching behind until about midlength (♂) or front third (♀) along telson. Paraterga 1–5 with two rather evident, setigerous indentations/spinules/knobs frontally and a short tooth posteriorly at base (Fig. 5A). Stricture between pro- and metazona very broad and shallow, always smooth at bottom (Fig. 5B, C). Pore formula normal; ozopores inconspicuous, located just at base on lateral side of poriferous paraterga. Transverse sulcus usually very vague, traceable in segments 4–18. Pleurosternal carinae evident only in segments 2 and 3 (Fig. 5A). Epiproct (Fig. 5C, D) rather simple, lateral pre-apical papillae very distinct, finger-shaped. Hypoproct (Fig. 5D) subtrapeziform, caudal margin emarginate, setigerous cones at caudal edge very large, widely separated. Axial line missing.

**Figure 6.**
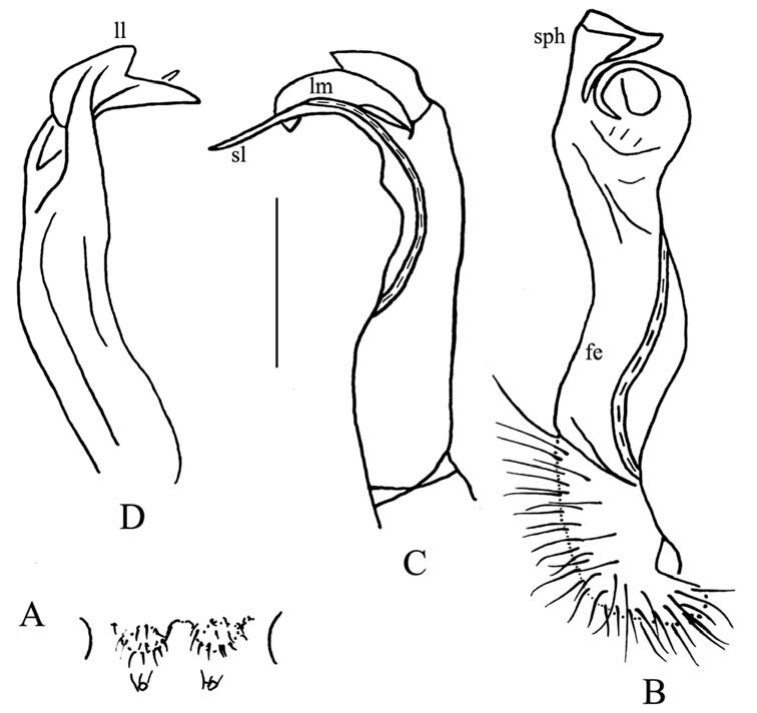
*Desmoxytes lui* sp. n., ♂ holotype. **A** sternal cones between coxae 4, ventral view **B–D** right gonopod telopodite, mesal, dorsal and sublateral views, respectively. Scale bar: 1.0 (**A**) and 0.5 mm (**B–D**). Designations: **fe** femorite **sph** solenophore **sl** solenomere **ll** lamina lateralis **lm** lamina medialis

Sterna quite densely setose, cross-impressions faint. A paramedian pair of entirely separated, short, rounded, setose tubercles between ♂ coxae 4 (Fig. 6A). Legs (Fig. 5A, B) extremely long and slender, ca 6.0 (♂) or 4.5 (♀) times longer than midbody height. ♂femora 6 strongly inflated ventrally in distal quarter (Fig. 5B).

Gonopods (Fig. 6B–D) simple. Coxite rather short, subcylindrical, poorly setose distodorsally, about 1/3 times as long as telopodite. Prefemoral portion (= densely setose) about half as long as acropodite. Femorite (**fe**) rather slender, elongate, only slightly enlarged distad, with seminal groove running entirely on mesal face, also with a distinct sulcus demarcating a short, strongly condensed solenophore (**sph**). The latter distinguished by a somewhat folded lamina medialis (**lm**) and a simpler lamina lateralis (**ll**). Solenomere (**sl**) similarly short, flagelliform, evidently separated at base from solenophore.

#### A key to *Desmoxytes* species currently known to occur in China

**Table:** 

1	At least paraterga on collum and following four segments spiniform, mostly very long and directed evidently more dorsally than laterally (Figs 3, 5). Guangxi Province	2
–	Paraterga wing- (Fig. 1) or antler-shaped	7
2	Paraterga long and spiniform only on collum and following four segments, evidently shorter on segment 5, small and coni- to tuberculiform thereafter (Fig. 5)	*Desmoxytes lui* sp.n.
–	Paraterga subequally long and spiniform at least in segments 2–18 (Fig. 3)	3
3	Both ♂ femora 6 and 7very evidently humped ventrally in distal quarter (Fig. 3E)	*Desmoxytes spinissima* sp. n.
–	Either ♂ femora 6 or 7 very evidently humped distoventrally	4
4	Only ♂ femora 7 very evidently humped distoventrally. Gonopod prefemoral portion less than 1/3 as long as acropodite	*Desmoxytes longispina*
–	Only ♂ femora 6 very evidently humped distoventrally. Gonopod prefemoral portion more than 1/3 as long as acropodite	5
5	Paraterga of collum rather short spinules, much shorter than following paraterga	*Desmoxytes cornutus*
–	Paraterga of collum about as long as following ones	6
6	Only collum and following metaterga 2–4 with rather evident setigerous spinules arranged in three transverse rows, following metaterga devoid of such spinules	*Desmoxytes scutigeroides*
–	Collum and all following metaterga each with two transverse rows of spinules/setae	*Desmoxytes minutubercula*
7	Paraterga antler-shaped, evidently branched	*Desmoxytes draco*
–	Paraterga wing-shaped (Fig. 1A–E)	8
8	Paraterga long and mostly subfalcate (Fig. 1A–E)	*Desmoxytes eupterygota* sp. n.
–	Paraterga suberect, evidently stouter	9
9	Metaterga 9–19 each with four transverse rows of 3(4)+(3)4 setigerous spinules. Gonopod telopodite subfalcate	*Desmoxytes scolopendroides*
–	Each postcollum metatergum with only two transverse rows of 2(3)+2(3) setigerous spinules. Gonopod telopodite suberect	*Desmoxytes planata*

### *“Desmoxytes” philippina* and its true affinities

As we have noted above, spiniform paraterga must have appeared independently in several paradoxosomatid lineages, including the subfamily Australiosomatinae. This latter group is still rather vaguely defined as opposed to the other two subfamilies, Alogolykinae and Paradoxosomatinae, showing no clear-cut apomorphies. Like Alogolykinae, and contrary to Paradoxosomatinae, most of the Australiosomatinae are supplied with adenostyles (= ventral projections) on ♂ femora 1, coupled with the gonopod showing a shorter or longer prefemoral portion, and a usually (but not always) shortened femorite crowned with one to several processes at or near the base of a strong solenomere. Contrary to the conditions observed in the other two subfamilies, this strong, thick, often apical solenomere in Australiosomatinae requires no or almost no support from the neighbouring structures, thus never being set off at or near the distal end of the femorite by a lateral sulcus or cingulum to develop a special sheath in the form of a solenophore. A complex, often membranous, apical solenophore that is clearly set off at least by one sulcus or cingulum at or near the distal end of the femorite must have appeared later in order to protect and sheath a mostly thin, weak, flagelliform solenomere.

In short, we see the main evolutionary trend in the family Paradoxosomatidae as the progressive development of a complex solenophore in conjunction with the solenomere’s growing flexibility and fragility. The Australiosomatinae would therefore be the basalmost group, with the Alogolykinae (and probably also a few Paradoxosomatinae like the tribe Paradoxosomatini and a few genera of the tribe Chamberlinini which also have solid solenomeres – see Golovatch 2011, Nguyen Duc and Korsós 2011) likely forming the next step by still retaining a strong and thick solenomere, the apical part of which, however, is already protected and sheathed by a solenophore. Finally, in the bulk of the Paradoxosomatinae (and of the Paradoxosomatidae as a whole), a distinctly delimited, complex, mostly membranous solenophore has developed to sheath and protect the weak, flagelliform solenomere.

Jeekel (1968) divided the Australian Australiosomatinae into two tribes, Australio- somatini and Antichiropodini, seeing the main differences between them in gonopod structure alone. Thus, the gonopod in Australiosomatini was stated to show a short to longer prefemoral portion and a stout femorite with several strong branches, including a modestly developed solenomere. In contrast, Antichiropodini were said to be characterized through a short prefemoral portion, an elongated femorite and a similarly long, strong, apically or subapically located solenomere. Golovatch (1996), when treating several Australiosomatinae from Borneo, regarded *Euphyodesmus* Attems, 1931 and a newly described genus, *Borneochiropus* Golovatch, 1996, as particularly close to each other, having also transferred both to the tribe Antichiropodini. Not only do at least some of their constituent species show spiniform paraterga so vividly reminding of those observed in certain *Desmoxytes*, but both lack adenostyles while their gonopod traits are especially similar: the prefemoral portion is medium-sized to hypertrophied, accordingly the femorite is elongate to strongly reduced, and the solenomere is apical to subapical. Both *Euphyodesmus* and *Borneochiropus* could have as well been assigned to Australiosomatini, but their placement into the Antichiropodini was favoured because their gonopods looked far simpler than those of most of the Australiosomatini in having only uni- or biramous telopodites, and the solenomere invariably apical or subapical.

In other words, the choice was indeed quite arbitrary. There are a few transitional conditions in gonopod conformation to be observed within *Euphyodesmus* alone which make it difficult to unequivocally assign this genus to either of the tribes.

This background information is necessary to properly assess the identity of *Desmoxytes philippina*, described recently from the Philippines (Nguyen Duc and Sierwald 2010). That it is nothing else but an Australiosomatinae has already been noted elsewhere, albeit indirectly because the species had not been described yet (Golovatch and Stoev 2010). Moreover, the gonopod structure (a uniramous telopodite with the prefemoral, femoral and solenomere parts being subequal in length, the femorite also twisted so that the seminal groove runs mostly along its lateral face, the absence both of a solenophore and of its basal delimitation sulcus), the lack of adenostyles, as well as the spiniform paraterga strongly suggest affinities to the Bornean *Euphyodesmus* and *Borneochiropus*. The proximity of the Philippines to Borneo supports this view. In no way does *Desmoxytes philippina* resemble a species of Orthomorphini, a tribe in which the seminal groove always runs along the mesal side of the gonofemorite before passing onto a flagelliform solenomere sheathed by an evident apical solenophore, both latter structures being set off at their bases at least by one distinct sulcus or cingulum.

So we are forced to remove *“D”. philippina* from *Desmoxytes* and assign it provisionally to a still unclassified genus of Antichiropodini or Australiosomatini, placing it there together with the similar *Euphyodesmus* and *Borneochiropus*. For the time being, we refer to this species as *“Desmoxytes”* in quotation marks to emphasize its highly doubtful generic allocation. The latter is the more so strange as Nguyen Duc and Sierwald (2010) explicitly noted the above basic differences. Placing *philippinus* provisionally in *Euphyodesmus* would have been more logical at the subfamily level.

## Supplementary Material

XML Treatment for Desmoxytes

XML Treatment for Desmoxytes eupterygota

XML Treatment for Desmoxytes spinissima

XML Treatment for Desmoxytes lui

## References

[B1] Cook OF Loomis HF (1924) A new family of spined millipeds from central China. Journal of the Washington Academy of Sciences 14: 103-108.

[B2] Deharveng L Bréhier F Bedos A Mingyi T Youbang L Feng Z Wengeng Q Xuefeng T (2008) Mulun and surrounding karsts (Guangxi) host the richest cave fauna of China. Subterranean Biology 6: 75-79.

[B3] Enghoff H Sutcharit C Panha S (2007) The shocking pink dragon millipede, *Desmoxytes purpurosea*, a colourful new species from Thailand (Diplopoda: Polydesmida: Paradoxosomatidae). Zootaxa 1563: 31-36.

[B4] Golovatch SI (1996) The millipede family Paradoxosomatidae on Borneo, with contributions to the faunas of some other islands of the Sunda area (Diplopoda, Polydesmida). Revue suisse de Zoologie 103 (1): 151-193.

[B5] Golovatch SI (2011) New or poorly-known Oriental Paradoxosomatidae (Diplopoda: Polydesmida), XI. Arthropoda Selecta 20 (4): 259-266.

[B6] Golovatch SI Enghoff H (1994) Review of the dragon millipedes, genus *Desmoxytes* Chamberlin, 1923 (Diplopoda, Polydesmida, Paradoxosomatidae). Steenstrupia 20: 45-71.

[B7] Golovatch SI Stoev P (2010) New or poorly-known millipedes (Diplopoda) from Papua New Guinea, 3. Arthropoda Selecta 19 (3): 145-152.

[B8] Golovatch SI Geoffroy JJ Mauriès JP (2010) Two new species of the millipede genus *Desmoxytes* Chamberlin, 1923 (Diplopoda: Polydesmida: Paradoxosomatidae) from caves in southern China. Arthropoda Selecta 19 (2): 57-61.

[B9] Jeekel CAW (1968) On the classification and geographical distribution of the family Paradoxosomatidae (Diplopoda, Polydesmida). Academische Proefschrift, Rotterdam, 162 pp.

[B10] Jeekel CAW (1980) The generic allocation of some little-known Paradoxosomatidae from South-East Asia (Diplopoda, Polydesmida). Revue suisse de Zoologie 87: 651-679.

[B11] Mesibov R (2006) Dirt-encrusted and dragon millipedes (Diplopoda: Polydesmida: Paradoxosomatidae) from Queensland, Australia. Zootaxa 1354: 31-44.

[B12] Nguyen Duc A Korsós Z (2011) A revision of the millipede genus *Riukiupeltis* Verhoeff, 1939 (Diplopoda, Polydesmida, Paradoxosomatidae), with comments on the status of related species. ZooKeys 156: 25-40. 10.3897/zookeys.156.2009 PMC3253568 22303093

[B13] Nguyen Duc A Sierwald P (2010) A new dragon millipede (Polydesmida: Paradoxosomatidae: Orthomorphini) from the Philippines. The Raffles Bulletin of Zoology 58 (2): 173-177.

[B14] Nguyen Duc A Golovatch SI Anichkin AE (2005) The dragon millipedes in Vietnam (Polydesmida: Paradoxosomatidae, genus *Desmoxytes* Chamberlin, 1923). Arthropoda Selecta 14 (3): 251-257.

[B15] Penev L Agosti D Georgiev T Catapano T Miller J Blagoderov V Roberts D Smith VS Brake I Ryrcroft S Scott B Johnson NF Morris RA Sautter G Chavan V Robertson T Remsen D Stoev P Parr C Knapp S Kress WJ Thompson FC Erwin T (2010) Semantic tagging of and semantic enhancements to systematics papers: ZooKeys working examples. ZooKeys 50: 1-16. doi: 10.3897/zookeys.50.538 PMC3088020 21594113

[B16] Penev L Hagedorn G Mietchen D Georgiev T Stoev P Sautter G Agosti D Plank A Balke M Hendrich L Erwin T (2011) Interlinking journal and wiki publications through joint citation: Working examples from ZooKeys and Plazi on Species-ID. ZooKeys 90: 1-12. doi: 10.3897/zookeys.90.1369 PMC3084489 21594104

[B17] Stoev P Enghoff H (2011) A review of the millipede genus *Sinocallipus* Zhang, 1993 (Diplopoda, Callipodida, Sinocallipodidae), with notes on gonopods monotony vs. peripheral diversity in millipedes. ZooKeys 90: 13-34. doi: 10.3897/zookeys.90.1291 PMC3084490 21594105

[B18] Zhang CZ (1986) On the genus *Pratinus* and its two new species from China (Diplopoda: Paradoxosomatidae). Acta Zootaxonomica Sinica 11 (3): 253-257.

[B19] Zhang CZ Li ZY (1982). *Centrodesmus cornutus* sp.nov., ein neue Diplopodenart aus dem Süd-China (Paradoxosomatidae: Polydesmida). Acta Zootaxonomica Sinica 7 (1): 37-39.

